# Frequent microdeletions in conventional papillary thyroid carcinoma detected by high-density oligonucleotide microarrays

**DOI:** 10.1186/1471-2164-15-S2-P62

**Published:** 2014-04-02

**Authors:** Alaa Al-Ahmadi, Reem Alotibi, Maha Al-Quaiti, Fai Ashgan, Kothandaraman Narasimhan, Etimad Huwait, Mamdooh Gari, Mohammed Hussein Al-Qahtani, Jaudah Al-Maghrabi, Hans-Juergen Schulten

**Affiliations:** 1 Center of Excellence in Genomic Medicine Research, King Abdulaziz University, Jeddah, Kingdom of Saudi Arabia; 2 Department of Biochemistry, King Abdulaziz University, Jeddah, Kingdom of Saudi Arabia; 3 Department of Pathology, Faculty of Medicine, King Abdulaziz University, Jeddah, Kingdom of Saudi Arabia; 4 Department of Pathology, King Faisal Specialist Hospital and Research Center, Jeddah, Kingdom of Saudi Arabia

## Background

The valine to glutamate substitution at codon 600 in exon 15 of the BRAF gene (V600E) is the major driver mutation in papillary thyroid carcinomas (PTCs). Contribution of genomic gains and losses to onset and progression of PTC is far less known. We assessed genomic imbalances in PTCs by utilizing high-density oligonucleotide arrays.

## Materials and methods

We used SurePrint G3 human CGH+*SNP*, 2×400K, microarrays to assess gains and losses in 47 PTCs in comparison to male and female human reference DNA. Interpretation of results was accomplished by using the HG19 version of the design file and the default analysis method of the Cytogenomics 2.7 research software [1]. To compare BRAF mutant (BRAF^mut^) PTCs with BRAF wild type (BRAF^wt^) PTCs, the BRAF mutational status was established in 42 cases by direct sequencing the mutational hotspot region in exon 15 [2].

## Results

Whole chromosome/chromosome arm imbalances (e.g., -1p, -16q, -19) were only infrequently observed and one case was in the triploid stage. The predominant forms of imbalances were microdeletions that were in general more pronounced in both BRAF^mut^ PTCs (N=27) and BRAF^wt^ PTCs (N=15). These microdeletions, observed in ∽40% or more of the cases, include known and yet unknown thyroid cancer susceptibility genes, for example TAF12 & RCC1 (1p, 28.8~28.9 Mb) YY1AP1 (1q, 155.7 Mb), PRKCI (2q, 169.9 Mb), GSTM2P1 & RPF2 & GTF3C6 & CDK19 (8q, 110.9~111.1 Mb), RASSF3 & TBK1 (12q, 64,5~65.2 Mb), MDM2 & NUP107 & RAP1B (12q, ~69.0~69.2 Mb), BRCA1 & NAGLU (17q, 40.6~41.1 Mb), and CDH2 (18q, ~25.5 Mb). Microamplifications, observed in ∽30% or more of the cases, include genes as USH2A (1q, 216.5 Mb), CTNNA2 (2p, 79.9 Mb), CLSTN2 (2q, 139.9 Mb), MSR1 (8p, 16.0 Mb), and CASP12 (11q, 104.6 Mb). Number and extent of regions with SNP homozygosity varied widely between the cases.

## Conclusions

This is one of the first studies using high-density oligonucleotide arrays to survey chromosomal imbalances in conventional BRAF^mut^ and BRAF^wt^ PTCs enabling to detect microdeletions/microamplifications (usually < 1 Mb) affecting known or yet unknown genes related to thyroid cancer. Further studies have to reveal how the affected genes contribute to onset and/or progression of PTC besides the known implication of the BRAF gain-of-function mutation in this disease.

*This study was supported by King Abdulaziz City for Science and Technology* (*KACST*) *grants 09-BIO707-03 and 09-BIO820-03.*
